# Spatial distribution of citizen science casuistic observations for different taxonomic groups

**DOI:** 10.1038/s41598-017-13130-8

**Published:** 2017-10-16

**Authors:** Patrícia Tiago, Ana Ceia-Hasse, Tiago A. Marques, César Capinha, Henrique M. Pereira

**Affiliations:** 1 CE3C, Centre for Ecology, Evolution and Environmental Changes, Faculdade de Ciências da Universidade de Lisboa, 1749-016 Lisbon, Portugal; 2 Cátedra Infraestruturas de Portugal-Biodiversidade, CIBIO/InBIO, Universidade do Porto, Campus Agrário de Vairão, 4485-661 Vairão, Portugal; 3 German Centre for Integrative Biodiversity Research (iDiv), Halle-Jena-Leipzig, Deutscher Platz 5e, 04103 Leipzig, Germany; 4 Centre for Research into Ecological and Environmental Modelling, The Observatory, University of St Andrews, St Andrews, KY16 9LZ Scotland; 5 Centro de Estatística e Aplicações, Departamento de Estatística e Investigação Operacional, Faculdade de Ciências, Universidade de Lisboa, 1749-016 Lisboa, Portugal; 6 Global Health and Tropical Medicine, GHTM, Instituto de Higiene e Medicina Tropical IHMT, Universidade Nova de Lisboa, UNL, Rua da Junqueira 100, 1349-008 Lisboa, Portugal; 7 Institute of Biology, Martin Luther University Halle-Wittenberg, Am Kirchtor 1, 06108 Halle (Saale) Germany

## Abstract

Opportunistic citizen science databases are becoming an important way of gathering information on species distributions. These data are temporally and spatially dispersed and could have limitations regarding biases in the distribution of the observations in space and/or time. In this work, we test the influence of landscape variables in the distribution of citizen science observations for eight taxonomic groups. We use data collected through a Portuguese citizen science database (biodiversity4all.org). We use a zero-inflated negative binomial regression to model the distribution of observations as a function of a set of variables representing the landscape features plausibly influencing the spatial distribution of the records. Results suggest that the density of paths is the most important variable, having a statistically significant positive relationship with number of observations for seven of the eight taxa considered. Wetland coverage was also identified as having a significant, positive relationship, for birds, amphibians and reptiles, and mammals. Our results highlight that the distribution of species observations, in citizen science projects, is spatially biased. Higher frequency of observations is driven largely by accessibility and by the presence of water bodies. We conclude that efforts are required to increase the spatial evenness of sampling effort from volunteers.

## Introduction

Citizen science has become a relevant tool for collecting species data^1^. Observations gathered by a large number of volunteers, over broad spatial extents and temporal periods often provide a large number of records^2^, allowing studies that would otherwise be unfeasible. The increment of species data from citizen science initiatives in recent years, seems to be particularly important for taxonomic groups that were less usually targeted in traditional citizen science projects, which were directed to species groups more conspicuous and easier to identify. Groups such as invertebrates or aquatic organisms were traditionally less targeted, and have benefitted in recent years.

Emerging technologies are also changing the type of volunteers that get involved with scientific projects^3^. Web 2.0, characterized by greater user interactivity and collaboration, more pervasive network connectivity and enhanced communication channels, permits easy overcrossing of social, cultural, economic, and political boundaries, and, also the integration of local/traditional knowledge in these projects^4^. The possibility of collecting, through mobile applications with internet connections, georeferenced observations of the natural world (e.g., wildlife sightings) via interactive geovisualization interfaces (e.g., Google Maps, Google Earth, and Microsoft Virtual Earth) or the use of sensors in the mobile devices allowing to collect data from the environment like air quality or noise.

The data collected can have different applications, such as creating species distribution maps (e.g.^5^) or identifying a biological invasion (e.g.^6,7^). The identification of spatial biases in the sampling provided by citizen science projects is fundamental to interpret the outcomes obtained. Only taking these biases into consideration, such as the existence of under-sampled regions, we can turn the results found useful for supporting the adoption of conservation measures by decision makers^8,9^.

Understanding where volunteers of biodiversity recording are collecting their observations is fundamental for a sensible use of the data collected. These volunteers do not select their survey locations randomly, but most likely as a combined influence of a number of factors^10^ such as accessibility^11^, proximity to urban centres, topographic variation, time of the year, species richness^8,12^ or other geographical or physical characteristic. Therefore, these databases may incorporate an important spatial bias, with some areas almost not being surveyed, while others corresponding to “hotspots” of observations^13,14^.

Another potential source of bias is the taxonomic group being recorded. Observations tend to focus on certain groups, generally those that are more easily detected and identified, such as birds or butterflies, or even certain species within a group. Moreover, volunteers may not record all the species they observe either because they are not able to identify them, due to lack of taxonomic expertise^15^, or because they aim to register only those that are rare, without an interest in recording species that are common^16,17^.

These data also have the limitation of being presence-only. In such cases, the non-recording of a species in a certain location by volunteers may correspond to the true absence of the species, to the inability of the volunteer to observe it or, to the overall absence of recording efforts^10^.

In this work, we explore the relationship between physical and geographical variables such as land cover, road or path density, human population and altitude, and the distribution of species observations of different taxonomic groups, as recorded by volunteers. We use records from the BioDiversity4All database (www.biodiversity4all.org), a country-wide citizen science project in Portugal. We aim to understand how observations are distributed across the country, which factors drive their distribution, and what type of relationship (e.g. negative or positive) the different variables form with the distribution of observations for the different taxonomic groups.

## Materials and Methods

### Species and volunteer data

We used opportunistic species observations data retrieved from the BioDiversity4All web portal (http://www.biodiversity4all.org/), a Portuguese citizen science project connected to an international project based in the Netherlands, Waarneming international (http://www.observado.org/), and which is similar to citizen science biodiversity databases elsewhere such as iNaturalist (http://www.inaturalist.org/) or iSpot (http://www.ispot.org/). BioDiversity4All started in 2010 but volunteers could add historical data so there is information referring to previous years. We only used species occurrences that provided GPS derived geographical coordinates, - ranging from 1982 until August 2016. We gathered the species observation records by their taxonomic group. In total, we considered data for 8 taxonomic groups: (1) plants, (2) mushrooms, (3) birds, (4) amphibians and reptiles, (5) mammals, (6) butterflies, (7) moths, and (8) other insects. For each of these groups we summed the number of species observations made in each 5 × 5 km grid cell. We only considered records for mainland Portugal, due to the inability of obtaining data for some of the predictive variables (below) for insular regions. We also collected the number of volunteers and the number of observations that each registered in the website.

### Geographic data

We identified a total of eight spatially explicit variables that had a potential to explain variation in the distribution of species observations: percentage of cover by artificial areas, percentage of cover by agriculture and agro-foresty areas, percentage of cover by forest and natural and semi-natural areas, percentage of cover by wetland areas (all sourced by^18^), road density (paved roads; km/km^2^), paths and footpaths density (i.e., paths open to non -motorized vehicles, and paths used mainly or exclusively by pedestrians; km/km^2^) (sourced by^19^), human population density (individuals/km^2^; log-transformed)^20^, and altitude (m)^21^. We selected these geographical variables because they are presumably relevant in driving the spatial behavior of species observers^13–17^. All variables covered the extent of mainland Portugal, at a 5 km resolution and were processed in QGis^22^. We tested for redundancy among data in the variables by calculating pairwise Pearson correlation.

### Statistical analyses

Given the large number of grid cells without species observations, we used a zero-inflated negative binomial regression (ZINB) to identify the variables that were related to the spatial distribution of the observations. ZINB are a default choice to deal with overdispersed counts, and in particular under situations where there are more zeros than the ‘simple’ negative binomial model might reasonably cope with (e.g.^23^). The ZINB models were implemented in R^24^ using the package pscl^25,26^. We tested for the significance and type of relationship of the explanatory factors and the counts of species observations in each grid cell for each taxonomic group, and also for all groups combined. We have not accounted explicitly for spatial autocorrelation in our models^27^.

## Results

**Figure 1:**
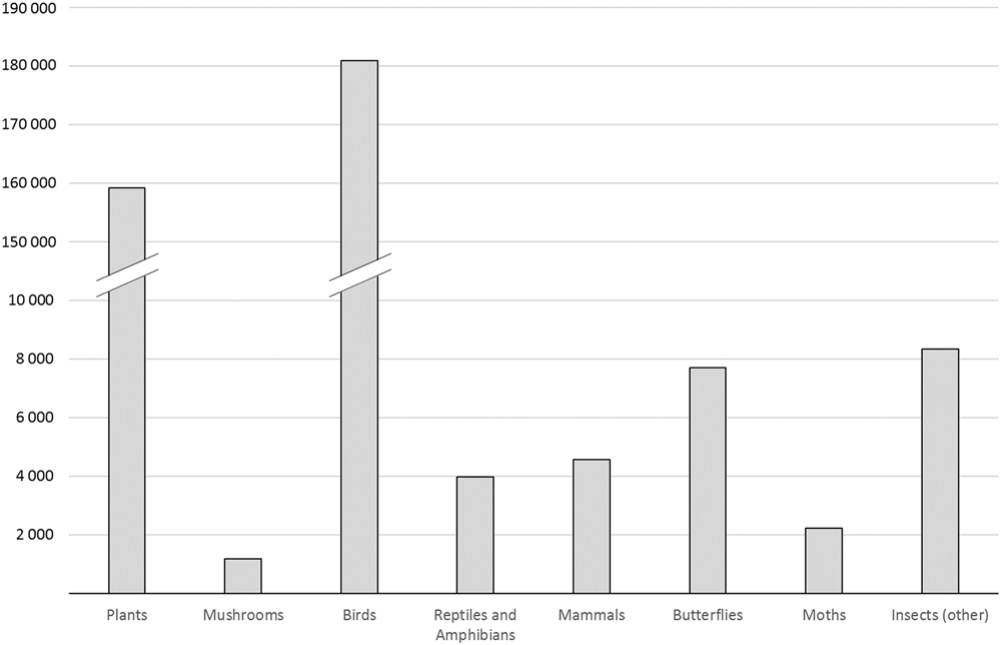
Number of citizen science observations registered in BioDiversity4All from May 1982 until August 2016 (*y*-axis) for each of the eight taxonomic groups analyzed (*x*-axis).

**Figure 2:**
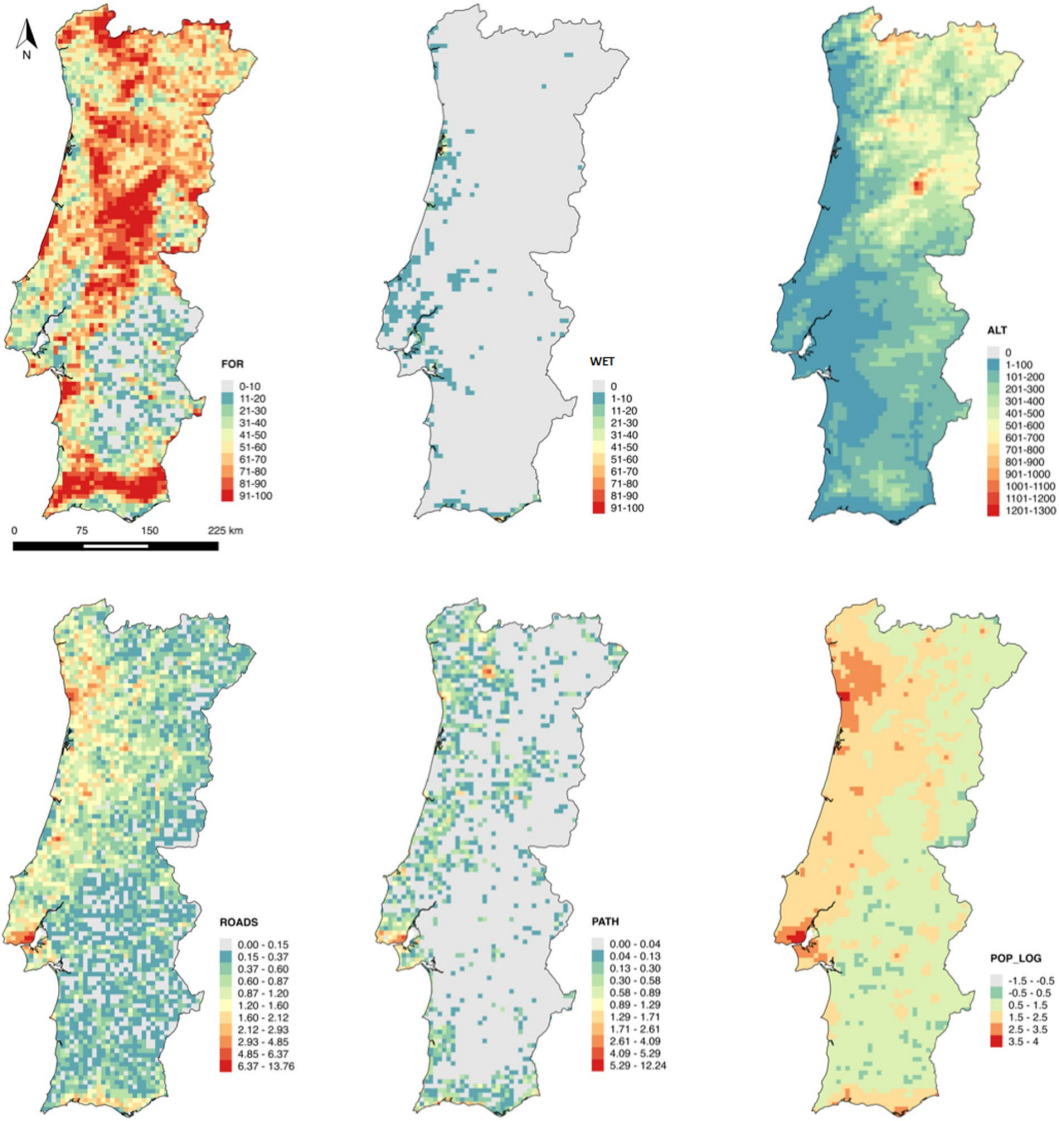
Explanatory variables tested for spatial association with the distribution of citizen science observations in mainland Portugal (FOR – percentage of cover of forest and natural and semi-natural territories, WET – percentage of cover of wetland territories, ROADS – density of roads, PATH – density of paths and footpaths, POP_LOG – logarithm of human population density, ALT – altitude). Figure created with QGis. 2014. Quantum GIS Geographic Information System. Open Source Geospatial Foundation Project. http://www.qgis.org/en/site/.

**Figure 3:**
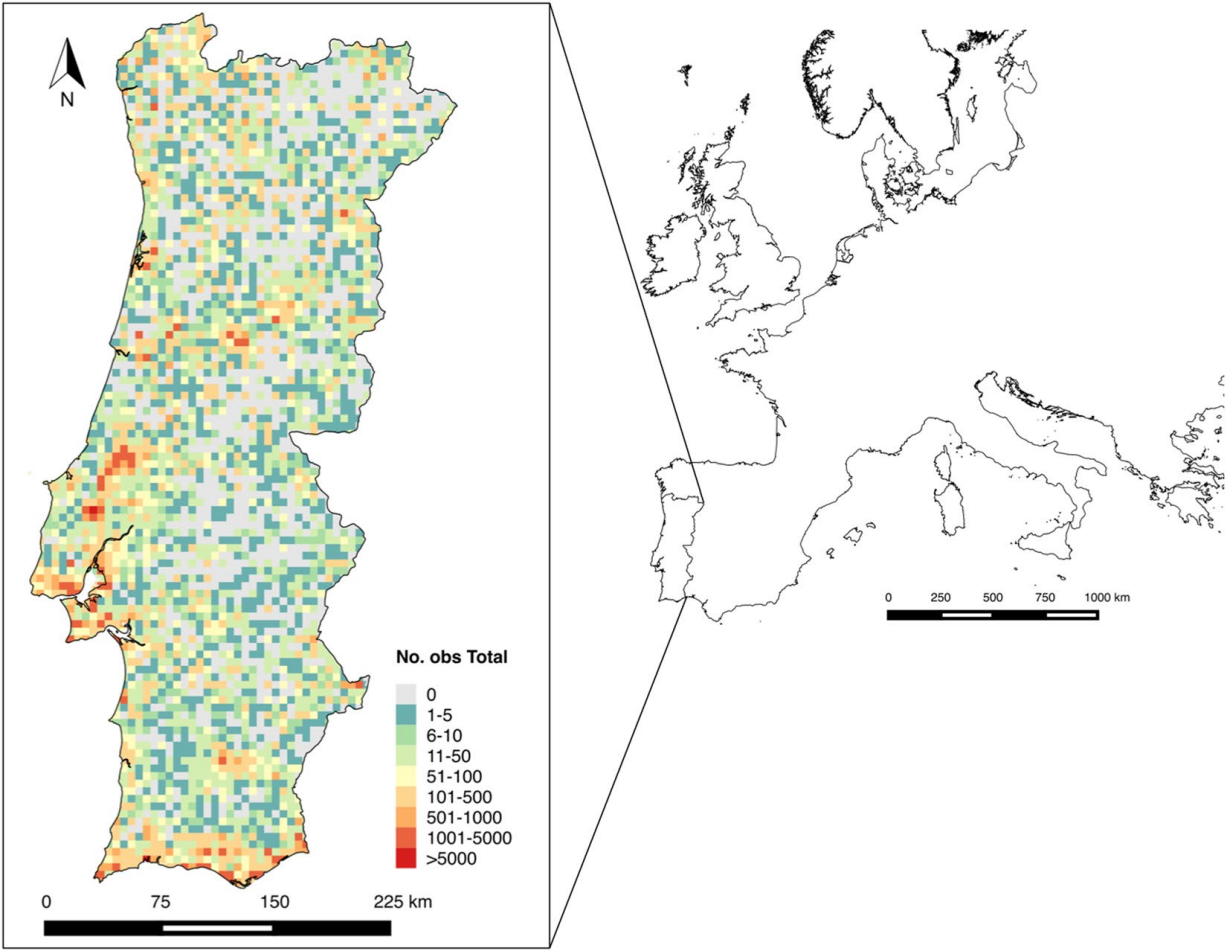
Location of the study area within Europe and total number of observations in mainland Portugal per grid cell. Figure created with QGis. 2014. Quantum GIS Geographic Information System. Open Source Geospatial Foundation Project. http://www.qgis.org/en/site/.

**Figure 4:**
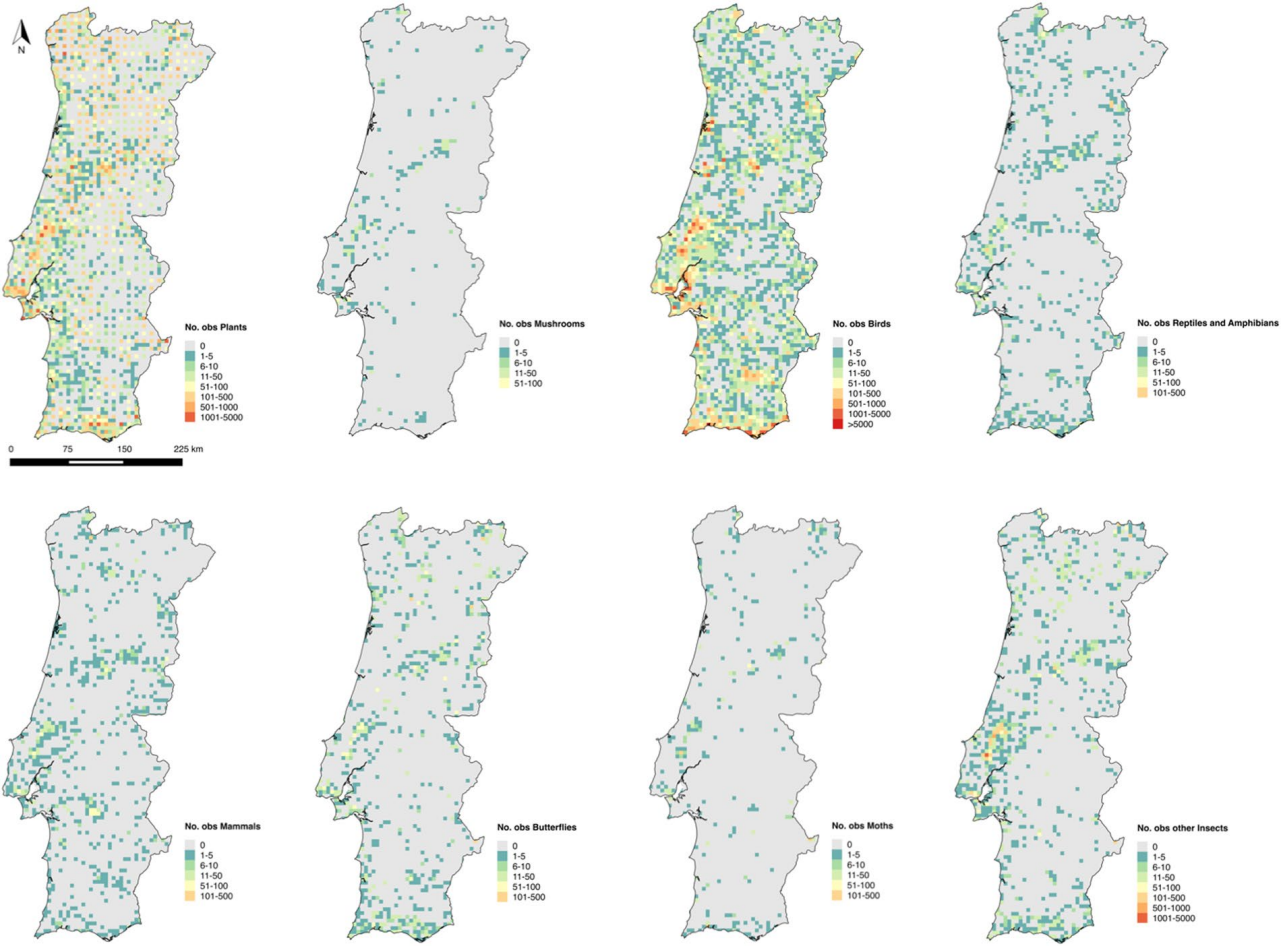
Number of citizen science species observations in mainland Portugal per grid cell, for each of the eight taxonomic groups analyzed. Figure created with QGis. 2014. Quantum GIS Geographic Information System. Open Source Geospatial Foundation Project. http://www.qgis.org/en/site/.

We adopted a spatial grid system where mainland Portugal comprises a total of 3 816 grid cells. The data compiled from Biodiversity4All included a total of 368 030 species observation records, from 1982 to 2016. Birds were the taxonomic group having the highest number of records, with a total of 180 911 records, followed by plants with 159 128 records. Mushrooms were the least recorded group having only 1 175 records (Fig. 1). The classes of explanatory variables for Portugal used in the analysis after being tested for redundancy are presented in Fig. 2. The mean number of records per grid cell is 88, and 1 030 cells have no observations (about 28% of the total area of mainland Portugal). The distribution of the number of records per grid cell for the different taxonomic groups, and for all groups combined, is shown in Figs. 3 and 4.

**Figure 5:**
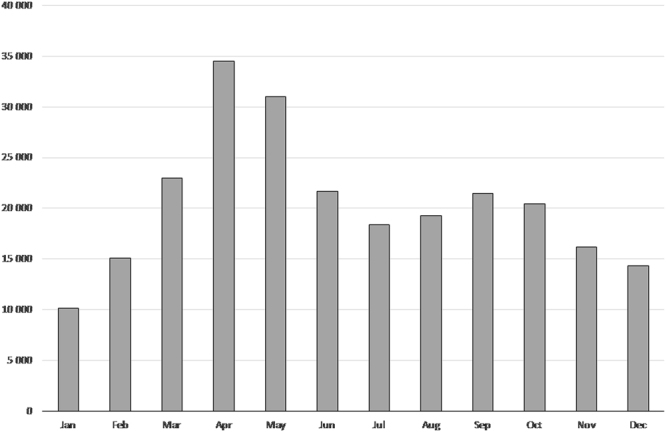
Total number of citizen science species observations (*y*-axis) made in each month from 2010 to 2015 (*x*-axis).

A temporal analysis of the data, for complete years (from 2010 to 2015), shows that April has the highest number of observations (34 497), followed by May (30 981) and by March (23 001) (Fig. 5).

**Figure 6:**
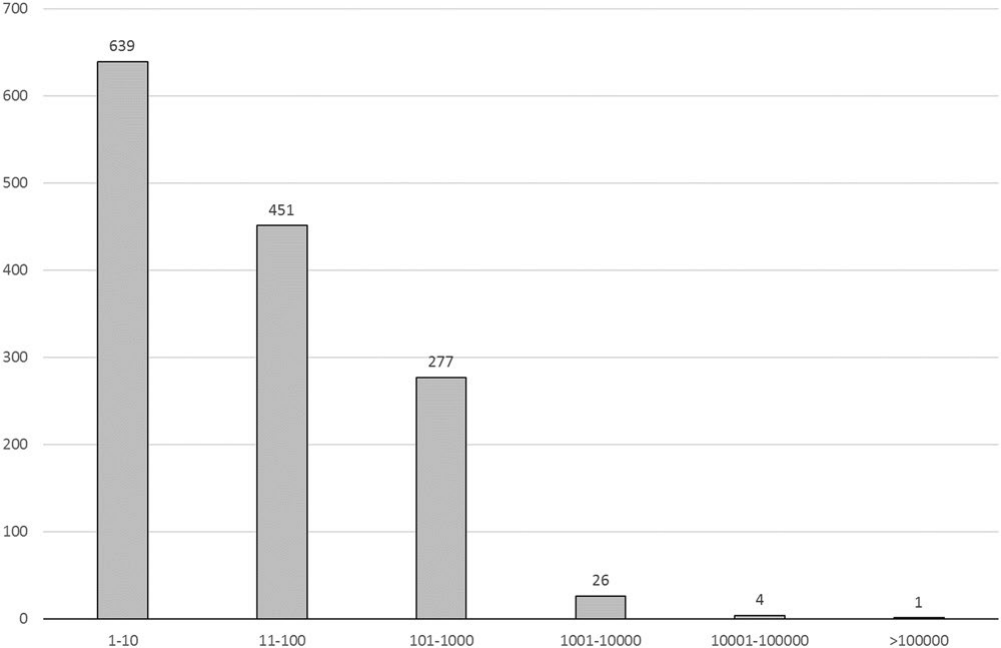
Number of volunteers (*y*-axis) grouped by level of species observations provided (*x*-axis).

**Figure 7:**
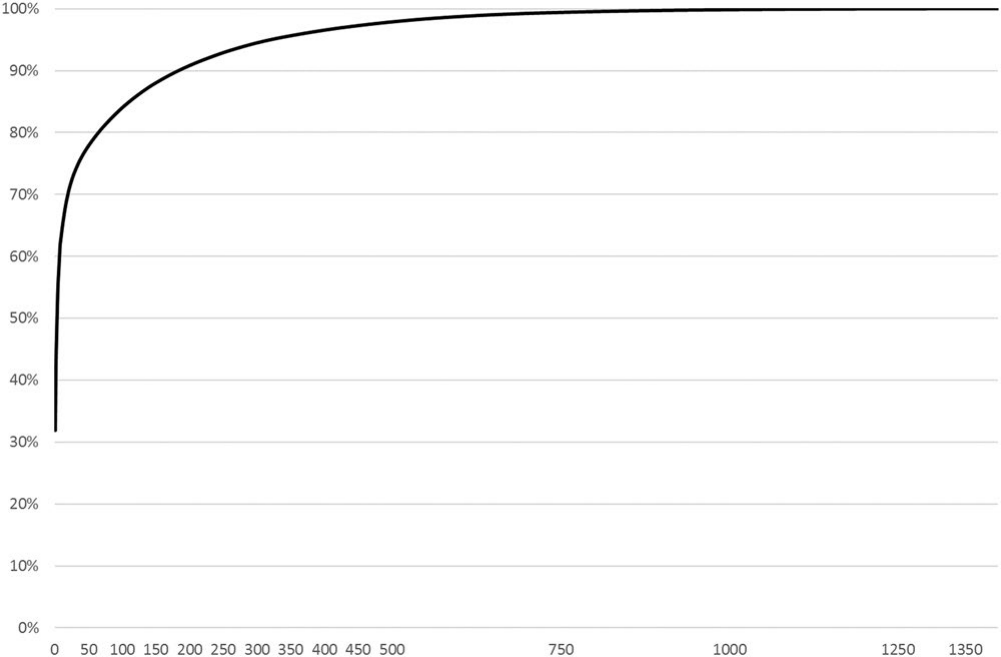
Cumulative number of species observations (*y*-axis) and the number of volunteers providing these observations (*x*-axis).

The total number of volunteers in BioDiversity4All, for the period considered, is 1 398. The number of volunteers with highest and lowest number of observations registered is shown in Fig. 6. The group of volunteers with 1 to 10 observations is the largest one with 639 people and only five volunteers recorded >10 000 observations. The number of volunteers responsible for 50% of the observations is 4 while 175 volunteers are responsible for 90% of the total amount of observations (Fig. 7).

We tested the correlation between the selected explanatory variables and excluded those that were highly correlated. In all cases, we kept the variables that we considered to provide a clearer link with causal mechanisms driving the behavior of observers. Hence, we excluded the percentage of cover by artificial areas, which was highly correlated with road density (Pearson correlation coefficient = 0.80, P < 0.05) and with logarithm of human population density (Pearson correlation coefficient = 0.71, P < 0.05). We also excluded the percentage of cover by agriculture or agro-foresty territories, which was highly negatively correlated with percentage of cover of forest and natural and semi-natural territories (Pearson correlation coefficient = −0.89, P < 0.05) (Table 1).

**Table 1:** Pearson correlation coefficients between the different explanatory variables: ART - percentage of cover of artificial areas, FOR – percentage of cover of forest and natural and semi-natural territories, AGR - percentage of cover of agriculture and agro-foresty areas, WET – percentage of cover of wetland territories, ROADS – density of roads, PATH – density of paths and footpaths, POP_LOG – logarithm of human population density, ALT – altitude.

Explanatory variables	ART	FOR	AGR	WET	ROADS	PATH	POP_LOG	ALT
ART	1.00							
FOR	−0.20	1.00						
AGR	−0.15	−0.89	1.00					
WET	0.07	−0.13	−0.07	1.00				
ROADS	0.80	−0.10	−0.15	−0.00	1.00			
PATH	0.46	0.02	−0.20	0.03	0.40	1.00		
POP_LOG	0.71	−0.09	−0.17	0.14	0.68	0.27	1.00	
ALT	−0.26	0.37	−0.23	−0.14	−0.13	−0.11	−0.16	1.00

Based on ZINB models we found that different explanatory variables relate to the distribution patterns of the observations for the different taxonomic groups (Table 2). Path density was the variable that most consistently explained the variation in the distribution of observations, being deemed as having a significant positive association in the models of 7 out of the 8 taxonomic groups considered (plants, birds, amphibians and reptiles, mammals, butterflies, moths, and other insects), as well as in the model for all the observations combined. The percentage of cover by forest and natural and semi-natural areas had a statistically significant positive relationship for plants, mushrooms, amphibians and reptiles, butterflies and other insects, as well as for the total number of observations. This was the second most important variable in the analysis. The logarithm of population density also showed a positive, statistically significant, relationship for plants, mushrooms, birds, other insects and the total observations. The percentage cover of wetland territories had a significant, positive relationship, for birds, and reptiles and amphibians. Finally, altitude had a statistically significant, negative relationship, with number of bird observations.

**Table 2:** Zero Inflated Negative Binomial Model (ZINB) relating the number of observations in each 5 × 5 km grid cells of Portugal (for total amount of observations and for each of the different taxonomic groups: plants, mushrooms, birds, amphibians and reptiles, mammals, butterflies, moths and other insects) and a set of variables (FOR – percentage of cover of forest and natural and semi-natural territories, WET – percentage of cover of wetland territories, ROADS – density of roads, PATH – density of paths and footpaths, POP_LOG – logarithm of human population density, ALT – altitude) (Level of significance *P < 0.05, **P < 0.01, ***P < 0.001).

Taxonomic Group	Model Summary	Variables						
		FOR	WET	ROADS	PATH	POP_LOG	ALT	Intercept
Total (all groups)	Model Coeficient	0.01	0.13	0.07	0.86	0.51	−4.05e-4	2.99
	Std Error	1.41e-3	0.02	0.08	0.12	0.08	1.80e-4	0.12
	Pr (>|z|)	2.62e-05***	1.71e-10***	0.41	4.70e-12***	3.42e-10***	0.02*	< 2e-16***
Plants	Model Coeficient	1.16e-2	4.50e-02	1.07e-01	5.49e-01	4.84e-01	−5.87e-05	2.00e + 00
	Std Error	2.22e-03	2.76e-02	1.23e-01	1.72e-01	1.25e-01	2.76e-04	1.85e-01
	Pr (>|z|)	1.76e-07***	0.10	0.38	1.41e-03**	1.11e-04***	0.83	< 2e-16***
Mushrooms	Model Coeficient	0.03	−0.12	−0.17	0.17	1.73	8.53e-04	−5.94
	Std Error	0.01	0.07	0.24	0.42	0.26	5.64e-04	0.40
	Pr (>|z|)	8.55e-09***	0.12	0.47	0.69	5.30e-11***	0.13	< 2e-16***
Birds	Model Coeficient	6.9e-04	0.17	0.13	1.30	0.34	−1.76e-03	2.69
	Std Error	1.61e-03	0.02	0.10	0.17	0.09	2.05e-04	0.14
	Pr (>|z|)	0.67	1.84e-11***	0.16	3.49e-15***	2.56e-04***	< 2e-16***	< 2e-16***
Amphibians and Reptiles	Model Coeficient	0.02	0.13	0.25	0.79	0.22	5.08e-04	−2.21
	Std Error	2.66e-03	0.03	0.14	0.21	0.14	3.46e-04	0.23
	Pr (>|z|)	5.00e-14***	1.14e-4***	0.06	0.40e-4***	0.11	0.14	< 2e-16***
Mammals	Model Coeficient	−2.12e-03	0.03	0.09	1.04	−0.13	9.86e-04	−0.40
	Std Error	2.23e-03	0.02	0.13	0.22	0.14	3.40e-04	0.12
	Pr (>|z|)	0.34	0.20	0.50	2.64e-06***	0.37	3.77e-03**	0.05*
Butterflies	Model Coeficient	0.01	0.08	0.43	1.31	0.06	1.71e-3	1.30
	Std Error	2.99e-03	0.03	0.17	0.29	0.16	3.77e-4	0.26
	Pr (>|z|)	6.02e-04***	0.02*	0.01**	4.97e-06***	0.70	3.77e-04	4.80e-07***
Moths	Model Coeficient	0.01	0.06	−0.20	2.84	0.02	1.50e-03	−1.97
	Std Error	0.01	0.06	0.32	0.66	0.32	6.79	0.40
	Pr (>|z|)	0.16	0.33	0.53	1.75e-05***	0.95	0.02*	6.61e-07***
Other Insects	Model Coeficient	0.02	0.01	−0.06	1.44	0.75	1.53e-04	1.40
	Std Error	2.81e-03	0.02	0.16	0.29	0.15	3.39e-04	0.20
	Pr (>|z|)	1.99e-08***	0.55	0.69	9.15e-07***	4.63e-07***	0.65	8.18e-13***

## Discussion

We quantified spatial recording of species observations, for 8 individual taxonomic groups and pooled across these, across mainland Portugal, and related these quantities to eight geographic variables likely to explain spatial variation in the number of observations. The interpretation of the results assumes that patterns found are mostly driven by changes in observer effort, either in space or across taxa, not by real differences in abundance/occurrence patterns for the taxa considered. This is a reasonable assumption provided the probability of detecting a given taxa in a given sampling unit is independent of the taxa abundance on that sampling unit. In other words, that all taxa considered and present in any given place would be detected by an observer. This seems reasonable at the coarse taxonomic level that the observations are made, which means that patterns found are either due to taxonomic differences (e.g. some observers prefer some taxa) or sampling differences (some areas are preferred by observers).

While we have not modelled explicitly spatial auto-correlation, we do not expect results presented to be sensitive to that choice. We therefore decided for this simple approach for the sake of pragmatism, avoiding the perhaps more elegant but necessarily more complex modelling approach, running the risk of obscuring the paper main messages.

A general characterization of our data shows that the distribution of records has a strong spatial bias, with areas of the country being highly covered while others having no observations, and that a limited number of volunteers are responsible for the majority of observations. The results also show strong seasonal patterns. This is not unexpected, since opportunistic citizen science databases are described as spatially and temporally biased^13,14^. The scarce number of volunteers responsible for a large proportion of the observations may be the main reason for this. In the case of this study, the reduced number of volunteers is also due to the lack of citizen science tradition in Portugal, leading to greater spatial data bias. It is also important to note that, for some specific taxonomic groups with different life histories, there are periods of the year when the groups/species can be observed and others when they cannot, or are more difficult to, such as hibernating reptiles, migratory species, and plants with different flowering periods.

Considering the variables that were identified to better explain the number of observations made, most of them indicate a positive effect of the accessibility of the survey area, such as altitude, density of roads (accessibility to a site - only found to be important for butterflies), or density of paths (accessibility within a site). Accessibility was already found to be important in determining where volunteers record observations^28,29^. Previous studies examining the spatial patterns of observations found strong roadside biases within woody plant records^30^, and have also showed that patterns differ between different taxonomic groups, such as between butterflies and mammals^29^.

Despite the variation between groups identified in the literature, we could identify some patterns across taxa. Path density showed a significant association with seven out of the eight taxonomic groups considered. In contrast with other studies^10^, density of paths explained more variation than the density of roads in taxa distribution records. Possibly these places also represent locations that people know will provide good outdoor walks and where it is easier to observe and identify species. While walking, volunteers have a higher availability to identify species and that is particularly important, for instance, for insects or plants that require a more detailed level of observation.

When considering the total number of observations, the group of birds and the group of amphibians and reptiles, the percentage of wetland areas also drives the frequency of observations. This can be explained by one or several different factors such as a higher attractiveness of these areas for the observers of a specific group (e.g., several birdwatchers go to wetland areas to observe birds, as these are ornithological-rich areas^31^), or by physiological characteristics of these groups, highly dependent of this type of habitat^32^.

It seems clear that analyzing patterns in volunteers’ distribution of observations is fundamental for planning different surveys that could help increase the data quality of these databases, and a better scientific use of the available information. Developing methods that evaluate and account for bias derived from different observation efforts (e.g.^12^) is a promising research topic and a good opportunity for collaboration between statisticians and conservation scientists, promoting the development of novel statistical approaches and survey designs^33^. In the absence of such approaches, at the very least the interpretation of such data must be made while considering the influence of the potential sources of bias. We note that the potential bias may be taxa specific, and its influence might change depending on the specific inferences being derived from the data. To conclude, with this work, we show that efforts are required to increase the spatial evenness of sampling effort in citizen science projects. That could be addressed with the use of additional incentive mechanisms or gamification baselines in order to increase sampling effort in some regions or for some taxonomic groups^34^.
